# Sphenoid Sinus Diseases: A Review of 1,442 Patients

**DOI:** 10.1155/2017/9650910

**Published:** 2017-09-27

**Authors:** Supranee Fooanant, Salita Angkurawaranon, Chaisiri Angkurawaranon, Kannika Roongrotwattanasiri, Saisawat Chaiyasate

**Affiliations:** ^1^Department of Otolaryngology, Faculty of Medicine, Chiang Mai University, Chiang Mai 50200, Thailand; ^2^Department of Radiology, Faculty of Medicine, Chiang Mai University, Chiang Mai 50200, Thailand; ^3^Department of Family Medicine, Faculty of Medicine, Chiang Mai University, Chiang Mai 50200, Thailand

## Abstract

**Objective:**

To review and report diseases of the sphenoid sinus from the literature and from a university hospital.

**Methods:**

Inpatients' data were retrospectively gathered and reviewed from January 2006 to June 2016. Clinical data, imaging, organisms, and pathological reports were collected. Pathology was divided into infection/inflammation, tumor, and miscellaneous. A literature review was performed with the search term “isolated sphenoid disease” in PubMed. Original primary studies with 20 patients or more were reviewed.

**Results and Discussion:**

One hundred and twenty-two patients were enrolled. Seventy-two subjects were female (59%). The average age was 54.3 years (±18.0). Imaging abnormalities were found incidentally in 27 patients (22.1%). The most common symptom was headache (63.9%). Visual loss, the second most common symptom, was more frequent in the tumor group (30.6% versus 54.2%). From the literature review, 21 primary studies with 1,320 total patients were included. From all studies and the present study, infection/inflammation was the most common pathology (75%) [95% confidence interval (CI): 0.696, 0.804]. Overall, tumors were found in 18.9% and malignant tumors in 7.0% [95% CI: 0.045, 0.095].

**Conclusion:**

A specific diagnosis of a sphenoid lesion is needed during active investigation. Infection/inflammation was the most common pathology and malignancy was found in 7%.

## 1. Introduction

The sphenoid sinus is situated at the center of the skull. Many vulnerable structures surround this sinus, for example, Proetz mentioned the dura mater, cranial nerves (III, IV, V1, V2, and VI), optic nerve and chiasm, internal carotid artery, cavernous sinus, pituitary gland, sphenopalatine ganglion, sphenopalatine artery, and pterygoid canal [1]. The symptoms are referred to these structures rather than involving the sinus.

Because of its deep-seated anatomy, this sinus does not usually present with nasal symptoms such as nasal obstruction or rhinorrhea. The most common symptom is headache and its prevalence ranges from 28% in tumor lesions to 98% in inflammatory lesions [2]. The next most common symptoms are cranial nerve deficit, visual alteration such as visual loss or diplopia, and pain or numbness according to trigeminal nerve involvement.

Nowadays, computed tomography (CT) and magnetic resonance imaging (MRI) are used to evaluate patients with suspected neurological problems; incidental abnormalities of the sphenoid sinus are noted for further management. This study aimed to summarize the diseases that occur in this area.

## 2. Materials and Methods

**Figure 1:**
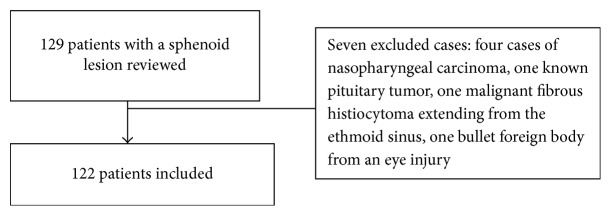
Study flow.

A retrospective study was performed in the Department of Otolaryngology, Chiang Mai University Hospital. Inpatients' data were gathered and reviewed from January 2006 to June 2016. Newly admitted cases of an isolated sphenoid lesion are identified in Figure 1.

The clinical presentation, nasal endoscopic results, imaging, organisms, and pathological reports were collected. Pathology was divided into infection/inflammation, tumor, and miscellaneous. Infection/inflammation consisted of sinusitis from bacterial, fungal, and other specific organisms; sphenoethmoidal polyps; retention cysts; mucosal hypertrophy; and mucocele. The tumor group included fibroosseous lesions, benign lesions, and malignant lesions, which were primary from the sphenoid or a metastasis. The miscellaneous group included cerebrospinal fluid (CSF) leakage, meningocele/meningoencephalocele, and vascular lesions such as pseudoaneurysm and others.

Imaging studies were performed using CT, MRI, or both. Abnormalities noted by radiologists included soft tissue or mucosal thickening, bone changes, content in the sinus, and enhancement, the details of which are not described in this present study.

A positive nasal endoscopy was defined as the presence of mucosal edema, pus, or tumor. The organisms were reported from aerobes and fungal culture.

A literature review was performed with the search term “isolated sphenoid disease” in PubMed on March 2017. Original primary studies with 20 patients or more in English abstracts and their related references were reviewed and included in the group diagnosis. Studies of specific patient groups such as children or diseases were not included in the review.

This study was approved by the Research Ethics Committee, Faculty of Medicine, Chiang Mai University. The new informed consent was waived as it was a retrospective study that had no effect on patients' present condition.

## 3. Results

There were 122 patients included in the study. Seventy-two were female subjects (59%) and 50 were male subjects (41%). The average age was 54.3 years (±18.0) and ranged from 1 year to 87 years [95% confidence interval (CI): 51.0, 57.5]. A sphenoid abnormality was found incidentally in 27 patients (22.1%). The most common indication for imaging in this group was a neurological condition (18 cases); there were nine cases of alteration of consciousness and memory, four cases of cerebrovascular disease, two cases of severe persistent headache, two cases of optic neuritis, and one case of epilepsy. Other indications were an evaluation of hearing loss or vertigo (five cases), malignancy survey (three cases), and persistent pain in the preauricular area (one case). No patient in the incidental group had a malignant lesion in the sphenoid. The most common lesions were fungal balls (15 cases). Others included six cases of bacterial sphenoiditis, two cases of chronic invasive aspergillosis, two cases of resolved sphenoiditis, one case of fibrous dysplasia, and one case of a retention cyst. Among the cases of invasive* Aspergillus* sphenoiditis, one presented with an alteration of consciousness and was first diagnosed with aseptic meningitis with improvement before otolaryngologists were consulted. The other case had a middle cerebral artery (MCA) infarction, which was suspected from the underlying atrial fibrillation.

**Figure 2:**
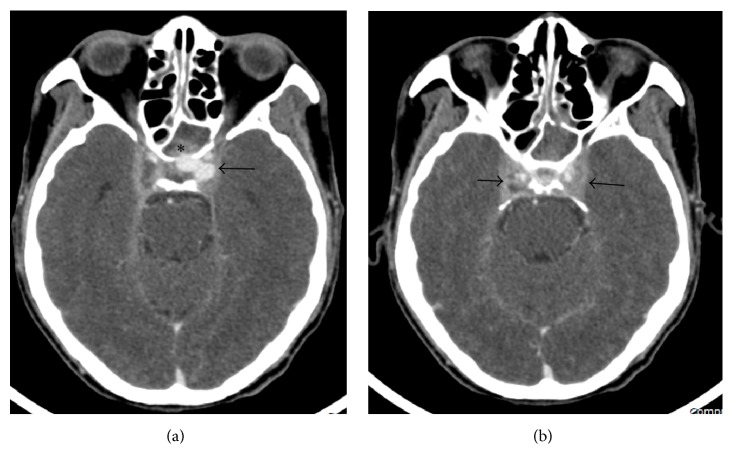
Total opacification of the left sphenoid sinus (*∗*) consistent with sinusitis. An infective pseudoaneurysm (arrow) from the cavernous segment of the left ICA protrudes into the left sphenoid sinus and sella turcica (a). Bulging with heterogeneous enhancement of the bilateral cavernous sinuses (arrows) represents thrombophlebitis (b).

The two cases of optic neuritis were documented as incidental findings because they were treated by the ophthalmologist and then scheduled for imaging. Their vision had improved by the time the imaging was acquired. One patient had a 5-mm supraclinoid internal carotid artery (ICA) aneurysm and sphenoiditis, which proved to be bacterial in nature and was at the site of the aneurysm (Figure 2). The other patient was treated by ophthalmologists with a systemic corticosteroid and MRI was performed during that period. Two months later, he was then sent to otolaryngologists for further management. Sphenoidotomy was performed but there was no discharge or mucosal swelling in the once-diseased sinus on MRI.

The spectrum of diseases is described in Table 1. Among the 98 cases of infection/inflammation, there were 40 cases of complicated sphenoiditis. Complicated bacterial sphenoiditis was present in 23.5% and complicated fungal sphenoiditis was present in 14.3%.

The most common symptom was headache, which was found in 78 patients (63.9%) (Table 2). Most described it as a hemicranial headache (48/78). Visual loss was the second most common symptom found in 35.3% and more commonly in the tumor group (30.6% versus 54.2%). The symptoms that were significantly more common in the infection/inflammation group than in the tumor group were rhinorrhea (*p* = 0.006) and fever (*p* = 0.012).

A pus culture was obtained from 76 patients (62.3%). Thirty-four patients (44.7%) had a single organism and 13 patients (17.1%) had multiple organisms on the culture report. The three most common bacteria were* Staphylococcus *spp. (27 patients),* Pseudomonas *spp. (14 patients), and* Klebsiella *spp. (six patients).

In the 48 patients with fungal sphenoiditis, bacterial growth was found in 19 patients (39.6%). The two most common organisms were the same as in the total culture reports.

### 3.1. Literature Review

From the “isolated sphenoid disease” search term, 145 articles were found in PubMed. After title and abstract screening and a cross-reference search, 21 primary studies (1,320 patients) were included in the analysis [1, 3–22] (Table 3). The study by Pearlman et al. [23] was not included because the patients were included in the study by Lawson and Reino [11] as well.

**Figure 3:**
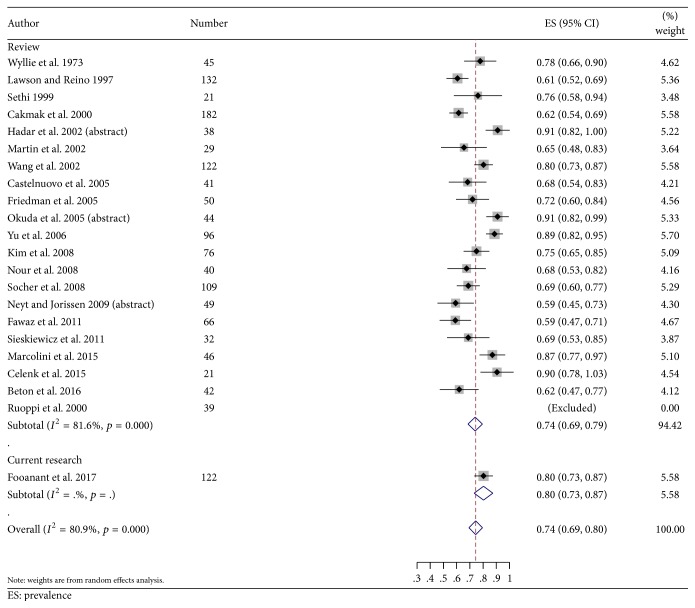
A forest plot shows the prevalence of infection/inflammation among the sphenoid sinus lesions.

**Figure 4:**
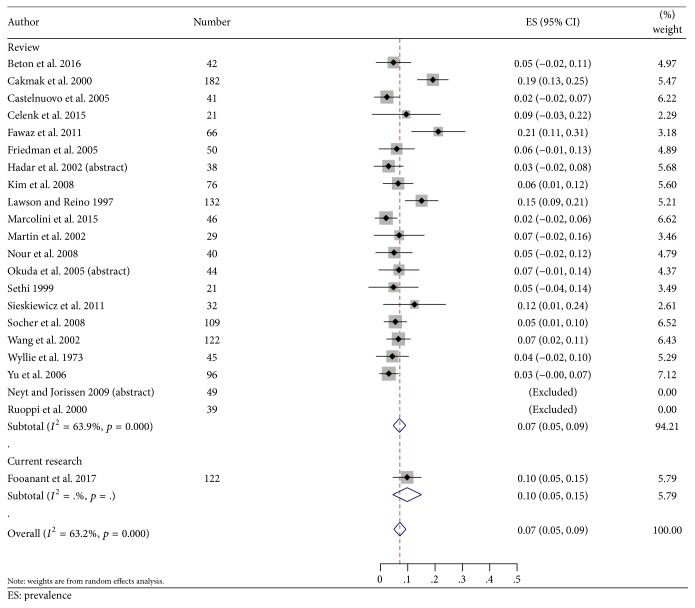
A forest plot shows the prevalence of malignant tumor among the sphenoid sinus lesions.

From all studies reviewed and the present study (Tables 1 and 3, Figure 3), infection/inflammation was the most common pathology (75%) [95% CI: 0.696, 0.804]; bacteria, fungus, mucocele, retention cyst, and polyp were included in this group and are stated in the order of their frequencies. Some studies classified fibroosseous lesions into the miscellaneous category and some included them in the tumor category with no detailed data; however, in this review, the authors classified them as tumors. Overall, tumors were found in 18.9% [95% CI: 0.145, 0.232] (Table 3) and malignant tumors were found in 7.0% [95% CI: 0.045, 0.095] (Figure 4). The frequency of fibrous dysplasia ranged from 1.5 to 13.1% while that of ossifying fibroma ranged from 1.5 to 2.4%. The miscellaneous group included 6.1% [95% CI: 0.034, 0.086]. The last group included a rhinolith, foreign body, CSF leakage, meningocele/meningoencephalocele, and aneurysm.

## 4. Discussion

Since the 1973 report by Wyllie et al. [1], the sphenoid sinus has remained a neglected area. It has no specific symptom of its own so the diagnosis requires a high index of suspicion and active processes of endoscopic examination, specific imaging, and/or surgical biopsy. Headache was the most common symptom in all studies. As the sphenopalatine ganglion and trigeminal nerve supply this area, the retroorbital, vertex, and occipital regions should be the major areas of complaint [11]. However, the most frequent area of headache varied in studies and included the retroorbital [1, 13, 20], frontal [4, 5], and vertex [6, 17] regions. Some studies mentioned a nonspecific headache area [10, 11, 21] as patients differently specified the headache location as diffuse [6] or pancephalic [8], hemicranial, periorbital, bifrontal, frontooccipital [6], parietal [5], temporal, or postauricular [15], and even in the maxillary area [8]. The high intensity and intractability made the attending physicians request imaging such as a brain CT scan. In this study, headache was the most common symptom and was present in 63.9%, as we also included incidental cases. Endoscopic examination in this study could demonstrate an abnormality in 38.5%, but this can be normal in 28.6% [6, 18] to 61.4% [20]. In other words, a normal endoscopic examination could not totally exclude disease of the sphenoid sinus.

Infection/inflammation was the most common pathology in all the studies including this study. The most common lesion in this group was bacterial sphenoiditis in the majority of studies [1, 3, 4, 7–11, 13–20, 22]. There are four studies that show different results: a mucocele in two studies [12, 21], fungal infection in the study by Castelnuovo et al. [5], and a polyp in the study by Celenk et al. [6]. Tumor was the second most common; fibroosseous lesions were included in this group in many studies, though some grouped them into the miscellaneous category [24]. The prevalence of malignant lesions in this review of a total of 1,442 patients was 7% [95% CI: 0.045, 0.095], which is close to the 10.9% reported in Knisely's systematic review of 1,215 patients [25].

Miscellaneous lesions, which were frequently a rhinolith in the past (3.8% [11] to 4.1% [1]), have changed to CSF leakage, which now ranges from 4.1% [14] to 17.1% [5]. The centers operating on skull base lesions find this more commonly, while at our center, this group of patients first visited neurosurgery, though some cases were operated on by a team of neuro-ENT surgeons.

What we would like to emphasize in this study is that an incidental finding of a sphenoid sinus abnormality requires a physician's attention, as two out of 27 of our cases turned out to be chronic invasive fungal infections. These patients needed specific antifungal therapy and surgical removal.

There were cases in which the sphenoid sinus was small, of the concha or presellar type, and could not be seen clearly using the soft-tissue window on CT scans. Later, patients with headache developed complications such as a neurological deficit or visual abnormalities. These can be seen in our study and there are many reported cases in the literature, so we would like to urge involved doctors in every specialty to carefully review their patients to diagnose and treat them properly.

The limitation was that not all the cases which first started in neurosurgery clinic were in this study. But we could see the sphenoid diseases scope from the literature search.

## 5. Conclusion

Lesions of the sphenoid sinus can be found early with neuroimaging, though a specific diagnosis requires an active process of examination, specific imaging, or surgery. Infection/inflammation was the most common pathology and malignancy was found in 7%.

## Figures and Tables

**Table 1:** Diagnoses of sphenoid lesions.

Group diagnosis		Frequency (%)	Detail
Infection/inflammation	Bacterial sphenoiditis	40 (32.8%)	One with a supraclinoid ICA aneurysm
	Fungal sphenoiditis	48 (39.3%)	37 fungal balls, 11 invasive
	Other inflammation	10 (8.2%)	Two resolved sphenoiditis, two chronic inflammation, two retention cysts, one mucocele, one polyp, one Tuberculosis (TB), one pseudoaneurysm^*∗*^
Tumor	Benign, benign fibroosseous lesion	12 (9.8%)	Three fibrous dysplasias, three pituitary adenomas, two meningiomas, two inverted papillomas, one giant cell tumor, one neuroendocrine tumor
	Malignant	12 (9.8%)	Two plasmacytomas, one myeloid sarcoma, one adenocarcinoma, one breast cancer, one melanoma, one neuroblastoma, one chordoma, one poorly differentiated carcinoma (CA), one squamous cell CA, one lymphoma, one neuroendocrine CA

^*∗*^This pseudoaneurysm case presented with clinical meningitis. On his brain CT scan, a pseudoaneurysm of the cavernous segment of the internal carotid artery (ICA) was found with an extension into the cloudy sphenoid sinus. After a 2-week course of intravenous antibiotics and surgical clipping of the supraclinoid ICA and bypass (common carotid artery-middle cerebral artery), his sphenoiditis resolved without surgical drainage.

**Table 2:** Characteristics of the infection/inflammation and tumor groups.

	Infection/inflammation 98 patients	Tumor 24 patients	*p*value^*∗*^
Mean age, years (SD) 95% CI	55.4 (±17.2) 51.98, 58.89	49.5 (±20.8) 40.66, 58.26	0.1463^*t*^
Female : male ratio	57 (58.2%) : 41 (41.8%)	15 (62.5%) : 9 (37.5%)	0.818
*Symptoms (% among all patients)*			
Headache (63.9%)	63 (64.3%)	15 (62.5%)	1.000
Visual loss (35.3%)	30 (30.6%)	13 (54.2%)	0.055
Facial pain (31.2%)	24 (24.5%)	6 (25.0%)	1.000
Rhinorrhea (24.6%)	36 (36.7%)	2 (8.3%)	0.006
Diplopia (18.9%)	17 (17.4%)	6 (25.0%)	0.392
Fever (16.4%)	20 (20.4%)	0	0.012
Meningeal sign (4.9%)	6 (6.1%)	0	0.597
Positive nasal endoscopy (38.5%)	39 (39.8%)	8 (33.3%)	0.644

SD: standard deviation, CI: confidence interval; ^*∗*^exact test, *t*: *t*-test.

**Table 3:** Sphenoid sinus lesion studies.

	Author	Number of patients	Mean age, years	Infection/ inflammation	Tumor^*∗*^	Miscellaneous
(1)	Wyllie et al. 1973	45	48	77.8%	17.8%	4.4%
(2)	Lawson and Reino 1997	132	51	60.6%	31.8%	7.6%
(3)	Sethi 1999	21	47.7	76.1%	23.9%	0.0%
(4)	Cakmak et al. 2000	182	56.6	61.5%	34.6%	3.9%
(5)	Ruoppi et al. 2000	39	46	100.0%	0%	0%
(6)	Hadar et al. 2002 (abstract)	38		91.0%	9.0%	0%
(7)	Martin et al. 2002	29	52.3	65.5%	24.1%	10.4%
(8)	Wang et al. 2002	122	44.7	80.3%	13.1%	6.6%
(9)	Castelnuovo et al. 2005	41	45.3	68.3%	12.2%	19.5%
(10)	Friedman et al. 2005	50	51	72.0%	20.0%	8.0%
(11)	Okuda et al. 2005 (abstract)	44		91.0%	9.0%	0%
(12)	Yu et al. 2006	96	43	88.6%	10.4%	1.0%
(13)	Kim et al. 2008	76	45	75.0%	25.0%	
(14)	Nour et al. 2008	40	38.6	67.5%	20.0%	12.5%
(15)	Socher et al. 2008	109		68.8%	15.6%	15.6%
(16)	Neyt and Jorissen 2009 (abstract)	49		59.2%	34.7%	6.1%
(17)	Fawaz et al. 2011 (abstract)	66		59.0%	36.5%	4.5%
(18)	Sieskiewicz et al. 2011	32	44.3	68.8%	18.7%	12.5%
(19)	Marcolini et al. 2015	46		87.0%	6.5%	6.5%
(20)	Celenk et al. 2015	21	43.1	90.5%	9.5%	0%
(21)	Beton et al. 2016	42	40.7	61.9%	23.8%	14.3%
		1,320	46.5	74.8 %	18.9%	6.3%
	95% confidence interval			[0.691, 0.804]	[0.142,0.234]	[0.036, 0.090]

	This study	122	54.3	80.3%	19.7%	0%
	Fooanant et al. 2017					
	Total	1,442	46.9	75.0%	18.9%	6.1%
	95% confidence interval			[0.696, 0.804]	[0.145,0.232]	[0.034, 0.086]

^*∗*^Fibroosseous lesions were included in the tumor group.
